# Study on the Usefulness of APR Scores from the Viewpoint of Proinflammatory Cytokines

**DOI:** 10.1155/2015/981981

**Published:** 2015-01-15

**Authors:** T. Nakamura, D. Hatanaka, T. Yoshioka, S. Yamada, H. Goto

**Affiliations:** ^1^Division of Neonatology, Center for Maternal Fetal and Neonatal Medicine, National Hospital Organization Nishisaitama Chuo National Hospital, Tokorozawa-shi, Saitama-ken 359-1151, Japan; ^2^Division of Neonatology, Musashino Red Cross Hospital, Musashino-shi 180-8610, Japan; ^3^Shino-Test Corporation, 2-29-14 Oonodai Minami-ku, Sagamihara-shi 252-0331, Japan; ^4^Division of Pediatrics, Nagoya City West Medical Center, Nagoya-shi 462-8508, Japan

## Abstract

*Background*. Delayed diagnosis and treatment of newborn infection adversely impact outcomes. Clinical laboratory parameters have aimed to obtain the most correct and prompt diagnosis and treatment of this disease. This study simultaneously observed changes over time in APR as well as proinflammatory cytokines and anti-proinflammatory cytokines and aims to clarify usefulness of APR scores. *Methods*. We evaluated the usefulness of acute phase reactants (APR) in 46 newborns whose serum up to age 7 days had been stored, with comparison of three types (Group I: infection 15, Group F: fetal inflammatory response syndrome 17, and Group C: control 14) of APR-based scores, those of C-reactive protein (CRP), alpha1-acid glycoprotein (AGP), and haptoglobin (Hp), with proinflammatory cytokine levels. APR scores for CRP, AGP, and Hp and the levels of the proinflammatory cytokines IL-1*β*, IL-6, IL-8, IL-10, and TNF*α* were determined. *Results*. The cytokine levels started to increase from age 0 days and then decreased rapidly. The three APR scores, CRP, AG, and Hp, were elevated at age 0 days and then gradually decreased in infection (Group I) and fetal inflammatory response syndrome (Group F). The duration of antibiotic administration according to APR scores was significantly shorter in Group F than in Group I. *Conclusion*. This study demonstrated APR scores to be more useful for deciding whether antibiotics should be discontinued than proinflammatory cytokine levels.

## 1. Introduction

It is clear that delayed diagnosis and treatment of newborn infection adversely impact outcomes. Numerous studies on clinical laboratory parameters have aimed to obtain the most correct and prompt diagnosis and treatment of this disease. Some studies have focused on acute phase reactants (APR) and reported on a combination with high sensitivity/specificity or a combination of APR with cytokines, especially proinflammatory cytokines such as TNF*α*, IL-1*β*, IL-6, and IL-8, which stimulate APR production [1–4]. We clinicians believe that it is important for laboratory test results to be immediately accessible, that unnecessary procedures be avoided, and that the antibiotic dose administered during the treatment be appropriate in light of the clinical diagnosis. Goto first reported the usefulness of APR scores for the diagnosis of newborn infection approximately 35 years ago [5, 6]. During this period, Quick Turbo, which measures 3 types of APR, that is, AGP, CRP, and Hp, in approximately 3 minutes at the bedside, was developed. Unfortunately, its usefulness has not as yet been appropriately evaluated. This study simultaneously observed changes over time in APR as well as proinflammatory cytokines and anti-proinflammatory cytokines and aims to clarify usefulness of APR scores.

## 2. Methods and Materials

The subjects were 46 newborns who were admitted to the NICU/GCU of the Perinatal Center, National Hospital Organization Nishisaitama Chuo National Hospital, within 24 hours after birth during the 1-year period from January 1 through December 31, 2008. These newborns had been judged by attending physicians to require daily blood tests for close monitoring during the first week of life, and any serum remaining after these tests had been stored (in total, 438 newborns were admitted to our center during the 2008 calendar year). APR scores and measurements of the 3 types of APR were extracted from the examination results. The cytokine levels were measured using a cytokine assay kit, Q-Plex Inflammatory Array (QUANSYS Biosciences Inc., UT, USA). This assay kit can simultaneously measure 9 cytokines: IL-1*α*, IL-1*β*, IL-2, IL-4, IL-6, IL-8, IL-10, IFN*γ*, and TNF*α*. Nine distinct capture antibodies in each well of a 96-well plate were detected employing a CCD camera with solid-phase ELISA at Sagamihara R&D Center, Shino-Test Corporation.

The subjects were divided into 3 groups. Group I (infection) consisted of those who were clinically diagnosed as having an infection by attending physicians or whose pathogens were isolated by culturing blood and the infectious lesions. Group F (fetal inflammatory response syndrome: FIRS) consisted of those who presented with inflammation, but not infection, in the maternal uterus with infectious signs, such as a fever of 38°C, premature rupture of membranes, foul-smelling amniotic fluid, and chorioamnionitis or funisitis according to placental pathology. Furthermore, elevation of cytokine fetal plasma IL-6 to more than 11 pg/mL, as proposed by Gomez et al. [7], was applied as an additional criterion (cord blood was used instead of fetal blood). Group C (control) consisted of those whose APR scores did not indicate an infectious state (neither 3 points indicating an infectious state nor 2 points for being CRP- and AGP-positive) at any time during the first week of life. StatMate Ver. 4.01 was used for statistical analysis, and the Dunn method of Kruskal-Wallis was applied for the testing of independent groups. *P* value < 0.05 was considered statistically significant.

This study was approved by the Ethics Committees of the National Hospital Organization Nishisaitama Chuo National Hospital and written informed consent was obtained from the parent(s) or guardian(s) of the hospitalized newborns.

## 3. Results

There were 15 subjects in Group I (infection), including 1 with GBS sepsis, 1 with GBS pneumonia, 1 with MRSA sepsis, 4 with neonatal TSS-like exanthematous disease (NTED) caused by MRSA exotoxin, TSST-1, -2 with* Enterococcus faecalis* sepsis, 2 with pneumonia of unidentified pathogens, and 4 with bacterial infection of unidentified infectious lesions. The number of subjects in Group F was 17. Their APR values were sequentially measured, and antibiotics (ABPC 100 mg/kg/day and CTX 100 mg/kg/day) were administered until the absence of infection was confirmed, based on the results of various bacterial cultures on admission. Group C (control) consisted of 14 subjects not given antibiotics for infection during the first week of life. The gestational ages and birth weights of Group I, F, and C infants were 37.2 ± 2.9 weeks and 2452 ± 643 g, 38.3 ± 3.7 weeks and 2794 ± 628 g, and 35.1 ± 4.5 weeks and 2346 ± 893 g, respectively, with a significant difference in gestational age between Groups F and C (*P* = 0.0359). Antibiotic administration was started whenever their APR scores were 3 points, indicative of an infectious state, or 2 points due to being CRP- and AGP-positive, and was continued until the infections had resolved. For the subjects with sepsis, the administration duration was generally 7 to 10 days based on their APR scores.

**Figure 1:**
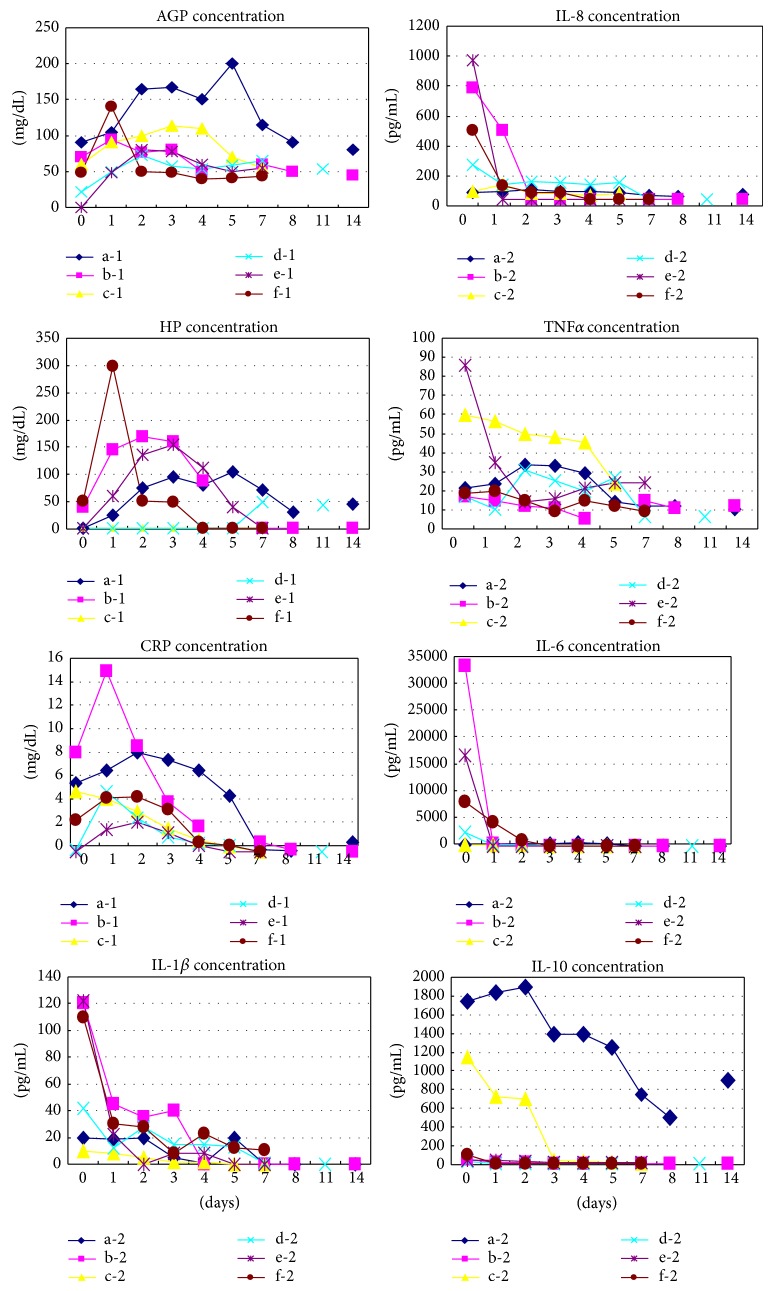
Changes over time in APR (CRP/AGP/Hp) and cytokine levels. (a-1) the levels of the APR in MRSA sepsis, (a-2) the levels of the proinflammatory cytokines in MRSA sepsis, (b-1) the levels of the APR in GBS sepsis, (b-2) the levels of the proinflammatory cytokines in GBS sepsis, (c-1) the levels of the APR in NTED, (c-2) the levels of the proinflammatory cytokines NTED, (d-1) the levels of the APR in FIRS-1, (d-2) the levels of the proinflammatory cytokines in FIRS-1, (e-1) the levels of the APR in FIRS-2, (e-2) the levels of the proinflammatory cytokines in FIRS-2, (f-1) the levels of the APR in FIRS-3, and (f-2) the levels of the proinflammatory cytokines in FIRS-3.

**Figure 2:**
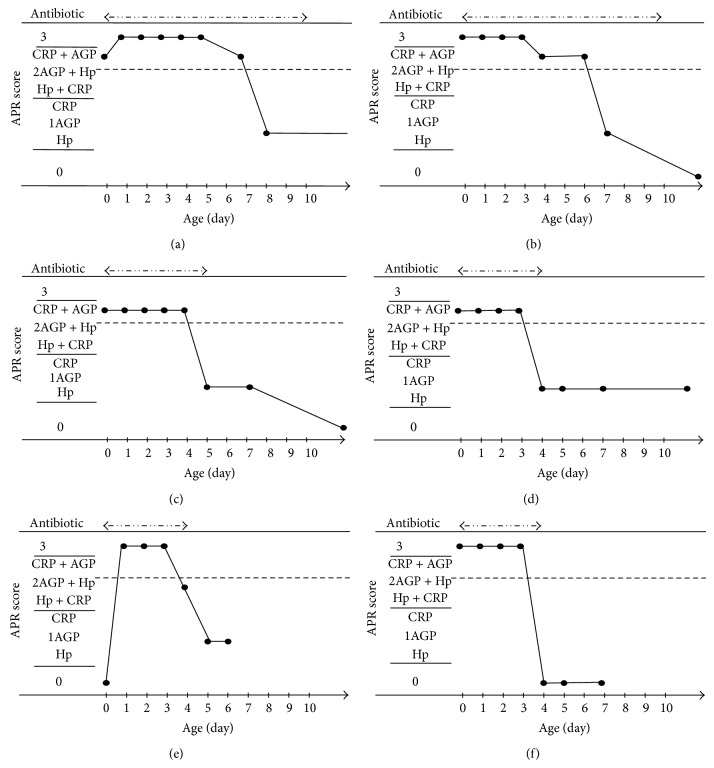
Changes over time in APR scores and antibiotic administration. (a) MRSA sepsis (b) GBS sepsis, (c) NTED, (d) FIRS-1, (e) FIRS-2, and (f) FIRS-3.

1. Comparisons among proinflammatory cytokine levels at age 0 days (Table 1): this measuring system evaluated differences in 4 proinflammatory cytokines, that is, IL-1*β*, IL-6, IL-8, and TNF*α*, at age 0 days among the three groups. IL-1*β* and TNF*α* levels were significantly higher in Group I than in Groups F and C. The IL-10 level was significantly higher in Group I than in Groups F and C.
2. Changes over time in APR (CRP/AGP/Hp) and cytokine levels (Figure 1): three subjects each from Groups I and F are illustrated. The levels of proinflammatory cytokines, including IL-1*β*, IL-6, IL-8, and TNF*α*, were higher from age 0 days in the Groups I and F subjects and subsequently decreased rapidly and then normalized, in most cases, in the early days after birth. APR increased over time from age 0 days and then, generally, showed a gradual decrease. In the subjects with MRSA infections in Group I, one of the anti-proinflammatory cytokines, IL-10, showed a remarkable increase from age 0 days as compared with the levels at the onset of other infections but subsequently showed a gradual decrease.
3. Changes over time in APR scores and antibiotic administration (Figure 2): as mentioned in (2), 3 subjects each from Groups I and F are illustrated. In Group I, the subjects with sepsis were administered antibiotics for 10 days, in principle, while in the others antibiotic administration was completed when the infectious state had resolved based on the changes over time in APR scores. The numbers of days of antibiotic administration in Groups I and F were 7.5 ± 2.9 days and 3.5 ± 1.3 days, respectively, indicating a significantly shorter administration period in Group F than in Group I (*P* = 0.007).

## 4. Discussion

The best method of diagnosing newborn infection is to detect the causative organism(s) as early and as accurately as possible. CRP, the main ARP, is increased not only by bacterial infection but also in the settings of various pathologies, for example, respiratory distress syndrome, asphyxia neonatorum, and meconium aspiration syndrome. Furthermore, CRP is produced and secreted by signaling hepatocytes in response to proinflammatory cytokines such as IL-1*β* and IL-6 and the lag time from the onset of infection until CRP elevation is 6 to 8 hours, possibly interfering with the usefulness of this measurement [1]. A recent report showed repeated measurements of both CRP and procalcitonin levels to have the highest sensitivity and specificity of currently available parameters for assessing response to treatment [2, 3]. Nonspecific and transient procalcitonin elevation was identified in early neonates, indicating that this level alone is more useful for diagnosing late-onset than early-onset newborn sepsis [4]. Furthermore, caution is warranted because procalcitonin is occasionally negative when inflammation is local, rather than the marked inflammation associated with sepsis. On the other hand, IL-6 increases only in the relatively early stages of infection and subsequently begins to decrease, within approximately 24 hours, indicating that IL-6 is suitable for diagnosing the earlier stages of infection but may not be an appropriate parameter for determining when to discontinue treatment. Furthermore, proinflammatory cytokine levels were often significantly greater in Group I than in Groups F and C, strongly supporting the usefulness of measuring these levels for assessing when to start antibiotic administration in newborns. However, the timing of the completion of administration was determined based on changes over time in APR scores and there was a significant difference in the number of dosing days between Groups I and F, which confirmed some of the advantages of using APR.

Laboratory tests cannot be used in clinical settings if the results cannot be obtained promptly. Proinflammatory cytokine levels increased during the early period of infection in this study, as expected from past observations. These measurement results are potentially very useful for initial diagnosis, if they can be obtained immediately, but the majority of institutions lack facilities for obtaining measurement results during routine clinical activities. In fact, measuring procalcitonin levels requires an automated immunofluorescence assay analyzer, such that only a few laboratories have the equipment necessary to perform this measurement [8].

On the other hand, Quick Turbo, a system independently developed by Shino-Test Corporation in Japan, can measure three APRs, CRP, AGP, and Hp, in as little as approximately 3 minutes. Furthermore, the sample serum volume is quite small requiring only 10 *μ*L for measurement of each of these 3 APRS (30 *μ*L in total). Thus, the necessary blood sample amount can be obtained even in very low-birth-weight infants, demonstrating that this device is quite useful in clinical settings. This device allows the diagnosis of infection using the scoring system proposed by Goto, whereby each APR is compared with the normal values by body weight and postnatal day and given a score (1 point for positive, 0 points for negative) and the scores for the three APRs are then totaled for a maximum of 3 points [5, 6]. According to our literature search, only a few reports have described diagnosing infections based on multiple APR measurements over time. In the 1980s, Bruns and Gahr measured CRP, *α*1-antitrypsin, and Hp over time but found only CRP to be useful, while the usefulness of the other 2 APR was unclear [9]. Philip reported that measurement over time of white blood cells as well as CRP, AGP, and Hp, as in the study by Goto et al., and mini-ESR were useful for diagnosing infection. At that time, a turbidimetric method was introduced which enabled a quantitative rather than a qualitative diagnosis to be obtained [10]. As reported by Goto, the diagnostic method employing the APR scoring system obtained with a bedside device, Quick Turbo, allowing immediate diagnosis of infection using small sample amounts, is a breakthrough [5, 6].

This study also confirmed that there is a time lag of 6 to 8 hours until APR becomes positive after infection onset as APR is a protein produced in the liver in response to stimulation with IL-1*β* and IL-6. Thus, the speed of diagnosis during the early stage of infection does not exceed that of the method using cytokines.

However, cytokines rapidly become negative and thus lose their usefulness for deciding when to discontinue antibiotic administration once such treatment has been started. It is more reasonable for the time point when the infectious state is no longer observed to be defined as the completion of treatment based on changes in APR scores over time, an approach which can be applied to determine the optimal duration of antibacterial agent administration.

Laboratory tests are indispensable for the diagnosis and treatment of newborn infection. However, a more important factor in clinical settings is for medical staff to consistently pay close attention to whether or not newborns develop signs and symptoms of infectious diseases. In other words, medical staff should have a comprehensive ability to determine at the bedside whether or not newborns are doing well and whether or not their risk of infection is high by focusing on the clinical course, which is ultimately the most important aspect of clinically managing newborns.

## Figures and Tables

**(a):** 

	*N*	IL-1*β*	IL-6	IL-8	IL-10	TNF*α*
Group I	15	151.2 ± 175.4	8402.1 ± 11784.7	25554.1 ± 34672.7	297.5 ± 551.4	72.2 ± 66.9
Group F	17	49.5 ± 46.4	16574.1 ± 38524.3	2511.2 ± 5226.8	58.1 ± 58.8	34.6 ± 31.6
Group C	14	4.6 ± 7.1	68.5 ± 140.3	85.2 ± 118.7	169.0 ± 507.0	8.4 ± 8.6

Mean ± SD.

**(b):** 

	*P*			*P*
I vs	Group F	Group C	F vs	Group C
IL-1*β *	*P* < 0.05	*P* < 0.01	IL-1*β*	NS
IL-6	NS	NS	IL-6	NS
IL-8	*P* < 0.05	*P* < 0.05	IL-8	NS
IL-10	NS	NS	IL-10	NS
TNF-*α*	*P* < 0.05	*P* < 0.01	TNF-*α*	NS

Group I: infection, Group F: fetal inflammatory response syndrome, Group C: control.

## References

[1] Chirico G., Loda C. (2011). Laboratory aid to the diagnosis and therapy of infection in the neonate. *Pediatric Reports*.

[2] Ng P. C. (2004). Diagnostic markers of infection in neonates. *Archives of Disease in Childhood: Fetal and Neonatal Edition*.

[3] Mishra U. K., Jacobs S. E., Doyle L. W., Garland S. M. (2006). Newer approaches to the diagnosis of early onset neonatal sepsis. *Archives of Disease in Childhood: Fetal and Neonatal Edition*.

[4] Ng P. C., Cheng S. H., Chui K. M. (1997). Diagnosis of late onset neonatal sepsis with cytokines, adhesion molecule, and C-reactive protein in preterm very low birthweight infants. *Archives of Disease in Childhood: Fetal and Neonatal Edition*.

[5] Goto H. (1988). The reliability for APR-Sc of newborn infection and clinical application in NICU. *Japanese Journal of Pediatrics*.

[6] Goto H. (2006). The reference acute phase reactants (APR) levels of newborn. *Neonatal Care*.

[7] Gomez R., Romero R., Ghezzi F., Mazor M., Berry S. M. (1998). The fetal inflammatory response syndrome. *American Journal of Obstetrics & Gynecology*.

[8] Kuroda S. (2010). Clinical usefulness of procalcitonin measurement. *The Journal of the Japanese Association for Infectious Diseases*.

[9] Bruns A., Gahr M. (1983). Sequential determination of CRP, *α*1-antitrypsin and haptoglobin in neonatal septicaemia. *Acta Paediatrica Scandinavica*.

[10] Philip A. G. S. (1984). White blood cells and acute phase reactants in neonatal sepsis. *Pediatrie*.

